# Effect of Porosity and Crystallinity on 3D Printed PLA Properties

**DOI:** 10.3390/polym11091487

**Published:** 2019-09-12

**Authors:** Yuhan Liao, Chang Liu, Bartolomeo Coppola, Giuseppina Barra, Luciano Di Maio, Loredana Incarnato, Khalid Lafdi

**Affiliations:** 1 Department of Chemical and Materials Engineering, University of Dayton, 300 College Park Drive, Dayton, OH 45469, USA; liaoy1@udayton.edu; 2 Department of Industrial Engineering (DIIN), University of Salerno, via Giovanni Paolo II, 132, Edificio E 84084 Fisciano, Italy; bcoppola@unisa.it (B.C.); gbarra@unisa.it (G.B.); lincarnato@unisa.it (L.I.); 3 Department of Mechanical Engineering, Northumbria University, Newcastle upon Tyne NE1 8ST, UK

**Keywords:** additive manufacturing, fused filament fabrication (FFF), semi-crystalline polymer, polylactic acid (PLA), porosity

## Abstract

Additive manufacturing (AM) is a promising technology for the rapid tooling and fabrication of complex geometry components. Among all AM techniques, fused filament fabrication (FFF) is the most widely used technique for polymers. However, the consistency and properties control of the FFF product remains a challenging issue. This study aims to investigate physical changes during the 3D printing of polylactic acid (PLA). The correlations between the porosity, crystallinity and mechanical properties of the printed parts were studied. Moreover, the effects of the build-platform temperature were investigated. The experimental results confirmed the anisotropy of printed objects due to the occurrence of orientation phenomena during the filament deposition and the formation both of ordered and disordered crystalline forms (α and δ, respectively). A heat treatment post-3D printing was proposed as an effective method to improve mechanical properties by optimizing the crystallinity (transforming the δ form into the α one) and overcoming the anisotropy of the 3D printed object.

## 1. Introduction

Additive manufacturing (AM), also called 3D printing, represents an emerging technique for building complex geometries and for rapid prototyping [1,2]. In the last 5 years, AM has rapidly evolved from the laboratory scale to the manufacturing of commercial parts [3]. Depending on the material and machine technology, there are several different processes to perform layer manufacturing. Among them, fused filament fabrication (FFF), selective laser sintering (SLS), stereolithography (SLA), laminated object manufacturing (LOM) and fused particle fabrication (FPF) are of relevant importance [1,2,3,4,5,6]. In particular, the FFF technique is the most used method for polymer-based modeling [7,8,9]. Although FFF of various polymers has already been commercialized, there is still the need to perform some fundamental studies to improve the process. For example, the development of porosity between the deposited filaments or the crystallization induced during the 3D printing process are critical issues that can compromise the quality of the final product, and hence it is extremely important to understand how to control these phenomena.

**Figure 1:**
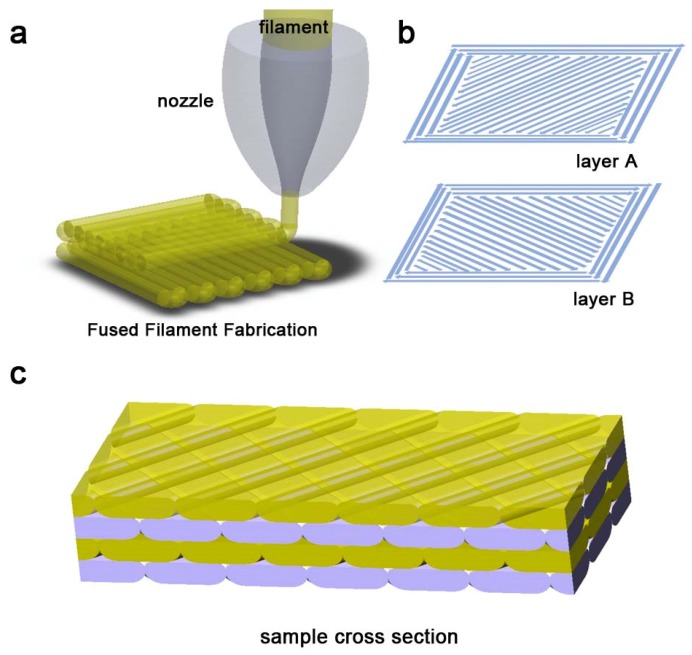
(**a**) An illustration of the FFF process and (**b**) samples printing pattern (±45°). (**c**) An illustration of the sample cross section.

In the FFF method, semi-molten polymer filaments are horizontally deposited. Usually, the polymer filament is driven into a heated channel (liquefier) by means of two grip-wheels. Then, the polymer melt is extruded out from a nozzle head and deposited onto a heated build-platform (Figure 1a). The printing conditions, such as the nozzle temperature, build-platform temperature, printing speed/pressure, nozzle movement speed and extrusion speed, influence the printed parts properties [10,11,12]. In particular, the printing temperature greatly influences the relaxation phenomena of polymer chains [13] and the layer adhesion both in terms of diffusion (i.e., the diffusion of polymer chains between two consecutive layers) and layer re-heating and welding [14]. Considering the layer thickness, a higher layer thicknesses leads to higher mechanical properties because layer coalescence is promoted and internal stresses are reduced [15]. However, layer thickness is also related to the surface quality of the 3D printed part (i.e., the surface texture and roughness).

Besides the instrumentation, the quality of the raw material also plays an important role. Considering the numerous printing parameters, optimal printing conditions are challenging and may require several iterations of trial and error corrections before printing appropriately [10,16], especially when the polymer is semi-crystalline. Many polymers used for FFF are semi-crystalline, such as polylactic acid (PLA) [17,18], polycaprolactone (PCL) [19], polybutylene terephthalate (PBT) [20], polyethylene (PE) [21] and polypropylene (PP) [22]. Although a wide range of polymer filaments are commercially available for FFF, PLA is the most popular one due to its degradability and availability in various colors and textures. However, the 3D printing behavior of pure PLA is still not well controlled due to its complex crystallization behavior [17,23,24]. Previous studies on FFF usually dismissed the crystallization process of PLA [17,18,23,24,25,26,27].

PLA exhibits various crystalline forms, and four main crystal forms (α, β, γ and δ) were mostly observed [28,29,30,31,32]. The common α crystallite form is obtained from the melt in a slow cooling procedure which allows the PLA chain to rotate into the confirmation with a lower potential energy. The β crystalline phase arises from a deformation of the α crystals and is usually obtained by drawing the PLA at elevated temperatures; the γ form is obtained by epitaxial crystallization on a single substrate such as hexamethylbenzene [32]. Due to the similarity in the crystalline structure, the δ form is also called α’ or imperfect α form [28,33]. The δ form (or α’ form) is observed in samples processed from the melt with a fast cooling procedure [32]. The crystallization of PLA at high super cooling of the melt leads to the formation of the disordered conformation, i.e., δ crystals. These crystals are metastable both at the temperature of their formation and below. From the melt, the polymer tends to crystallize in the α form but actually also partially crystallizes in the α’ form at lower temperatures [33]. In this case, the PLA has higher internal stresses and presents a lower thermal and mechanical stability [33]. Moreover, the printing process forces semi-molten PLA to flow through a narrow nozzle, and this process induces an orientation of the polymer chains as investigated in a previous study [13]. Considering the effect of the printing pathway as well, the printed part is always anisotropic [34]. Additionally, PLA is widely used as a polymeric matrix for nanocomposites and polymer blends. Thus, the crystalline behavior of PLA is further controlled by the properties of nano-fillers and its miscibility with other polymers [35,36]. Initially, many studies were focused on the use of polymer nanocomposites for several reasons, mainly the formation of conductive networks, the improvement of barrier properties and the possibility of fiber drawing [37,38,39,40]. As a consequence, the performance of 3D printed PLA nanocomposites was investigated [13,41]. It seems that the nano-filler induced crystallization of PLA could be an essential way to control the crystallization behavior of 3D printed PLA [42,43]. The anisotropy and mechanical properties of the printed item are strongly influenced by the printing parameters [13,26,44]. Moreover, considering that material fusion is essential in the FFF process, several scholars investigated the melting model and the flow during extrusion [45].

In this study, we examined the effect of the porosity and crystallinity of the printed parts. Moreover, a heat-treatment was proposed as a solution to improve the printed products’ mechanical properties.

## 2. Experimental Section

### 2.1. 3D Printing of PLA Samples

For the filament preparation, a PLA 4043D (NatureWorks, Minnetonka, MN, USA) was used. The glass transition temperature (T_g_), melting temperature (T_m_), D-isomer content and polydispersity index are 60 °C, 150 °C, 2% and 1.15, respectively. The PLA filaments were produced using a single screw extruder (Brabender Do-Corder E330, D_screw_ = 20 mm, L/D = 20) equipped with a capillary die of 3 mm, at a screw speed of 10 rpm, operating at the following temperatures: 180-180-160 °C (from hopper to die). The filaments were collected using a take-up system with air cooling in order to have a filament diameter of approximately 2.85 ± 0.10 mm.

To print the samples, a commercial 3D printer equipped with a nozzle of 0.35 mm (Ultimaker 3, Ultimaker, Utrecht, The Netherlands) was used. For the 3D printing, the nozzle temperature, layer height and raster angle were fixed and were equal to 180 °C, 0.1 mm and ±45°, respectively (Figure 1b).

Conversely, the effects of two different build-platform temperatures were investigated (40 and 80 °C, respectively). PLA filaments were used to print square specimens (50 × 50 mm^2^) of different thicknesses (e.g., 4 layers with a height of 0.36 mm, 24 layers with a height of 2.46 mm, approximately), previously designed using a CAD software. For a comparison, an injection molded sample was also prepared using the same PLA.

### 2.2. Sample Preparation

First, the sample cross section was polished for optical characterization. The samples were cut parallel to the sample border, 45° to the patterns (as shown in Figure 1c), and embed into epoxy resin. The cured epoxy blocks were then polished with a ceramic polishing solution (MasterPrep polishing solution, 50 nm, Buehler, Lake Bluff, IL, USA) using a Buehler AutoMet 250 polisher. After washing, the blocks were then quickly dried at room temperature.

For the mechanical test, the specimens were cut into designed geometries. In particular, the specimens for the TMA (Thermal Mechanical Analysis, Q400, TA instruments, New Castle, DE, USA) test were about 8 mm (length) by 5 mm (width) by 0.2 mm (thickness), while the specimens for the DMA (Dynamic Mechanical Analysis, Q800, TA instruments, New Castle, DE, USA) test were about 10 mm (length) by 8 mm (width) by 0.5mm (thickness). In order to distinguish the printed object properties at different heights (i.e., different distances between the build-platform), the specimens were carefully polished, dividing them in three parts: top, middle and bottom layers. Each specimen was controlled so as to have a thickness of 0.2 mm, approximately. For example, the top layers of thick specimens were prepared by grinding the middle and bottom layers out. The sample was grinded sequentially using different sand papers (240, 480, 600, 1500, and 2400 grit in sequence) and polished with the same polishing solution previously described. After a gentle washing procedure, the specimens were dried in a vacuum oven at room temperature. The heat treatment procedure on the 3D printed parts was carried out in a lab oven (Blue M, LO-225-P, Thermal Product Solutions, New Columbia, PA, USA) at 120 °C for 10 min. 

### 2.3. Characterization

#### 2.3.1. Optical Observation

The polished samples’ morphology was examined using an optical microscope (Axio, Vert A1, Zeiss, Oberkochen, Germany). In particular, the samples’ porosity was calculated based on cross section images, processed with ImageJ [46]. The specimens’ solidity (the complement of the porosity) was calculated according to the following equation (Equation (1)):

Solidity = (1 − Porosity) × 100%(1)

#### 2.3.2. Mechanical Testing

The 3D printed specimens were tested both in static and dynamic conditions. In the static-mechanical test, the specimens were tested using TMA with the tensile fixture with a preload of 0.1 N. Young’s modulus was calculated from the load-displacement curve considering the slope of the elastic part. In the dynamic-mechanical test, the specimens were tested using DMA with tensile fixture. The testing frequency was 1 Hz.

#### 2.3.3. X-ray Diffraction (XRD)

The orientation and crystalline forms of both the 3D-printed and annealed specimens were investigated by analyzing the wide angle X-ray diffraction (WAXD, Smartlab X-ray diffractometer, Rigaku, Tokyo, Japan) and 2-dimentional X-ray diffraction (2D-XRD, Oxford Diffraction Xcalibur 3 x-ray diffractometer) patterns. The 2D-XRD data were processed using the XRD2Dscan software (version 4.1.1, Universidad de Granada, Granada, Spain) [47].

## 3. Results and Discussion

### 3.1. Morphology

**Figure 2:**
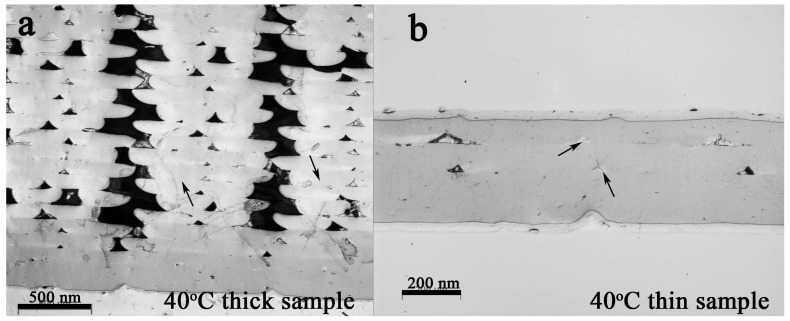
The cross section of the (**a**) thick and (**b**) thin PLA samples printed on the build-platform heated at 40 °C. The bar in (**a**) is 500 μm. The bar in (**b**) is 200 μm.

**Figure 3:**
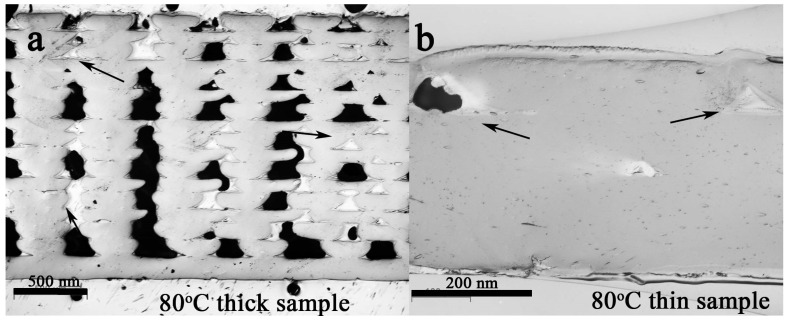
The cross section of the (**a**) thick and (**b**) thin PLA samples printed on the build-platform heated at 80 °C. The bar in (**a**) is 500 μm. The bar in (**b**) is 200 μm.

The cross section of the 3D printed samples was investigated to study the influence of the specimens’ thickness and build-platform temperature on the samples’ porosity. Figure 2 and Figure 3 show the cross section of the samples’ infill, oriented at ±45°, and several pores between the deposited filaments are clearly visible for both build-platform temperatures (40 °C in Figure 2, and 80 °C in Figure 3, respectively). As evident both in Figure 2 and Figure 3, filaments were deformed during the printing process (from circular to arched shaped) due to the nozzle contact with the semi-molten polymer because the fixed layer thickness was imposed as being 0.1 mm (having a nozzle diameter of 0.35 mm). Nevertheless, several voids were created between the deposited filaments.

It is noticeable that in both cases the first 3 layers are free of porosities compared to the middle and top layers. For a comparison, to better investigate the effect of sample thickness, 4-layer thin samples were printed as well (Figure 2b and Figure 3b). In both cases, only small triangular voids (indicated by the black arrows) can be observed.

To investigate the influence of the build-platform temperature, thick and thin samples were printed using a higher temperature, i.e., 80 °C. The cross sections of these samples are shown in Figure 3. Furthermore, in this case, several pores are particularly present in the thick sample (Figure 3a). Conversely, the thin samples (Figure 3b) have approximatively the same morphology as their analogous thick samples (Figure 2b), and in the case of both the thick and thin samples the bottom layers are less porous than the middle and top layers, as previously discussed. 

**Figure 4:**
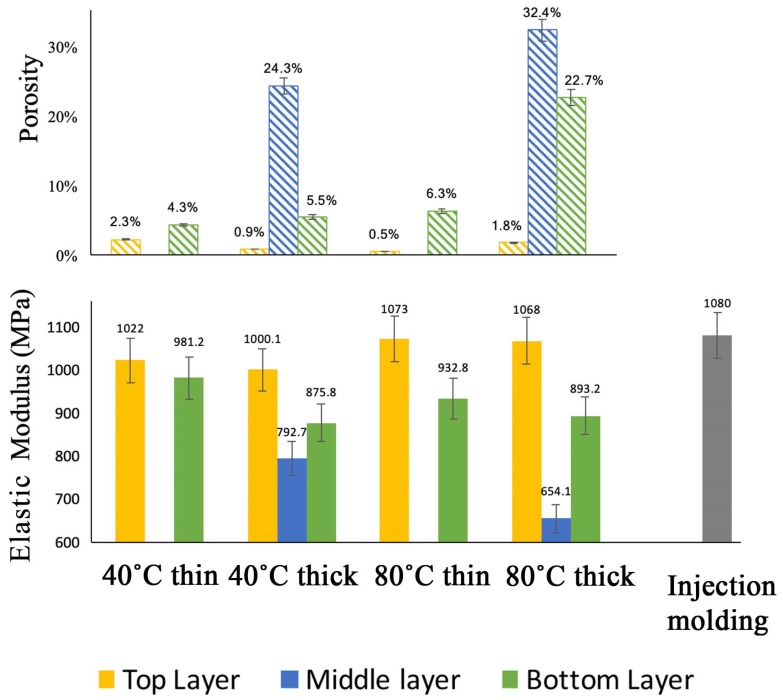
Porosity and elastic modulus of the samples.

The samples’ porosity was determined by processing the cross-section images with ImageJ, and the results are reported in Figure 4. It is evident that the porosity of the middle and top layers is higher than that of the bottom layers. Moreover, thick samples, whether the build-platform temperature are more porous than thin specimens. In particular, for 80 °C thick samples, the porosity of the middle layers is as high as 32% (Figure 4).

### 3.2. Mechanical and Thermo-Mechanical Properties

**Figure 5:**
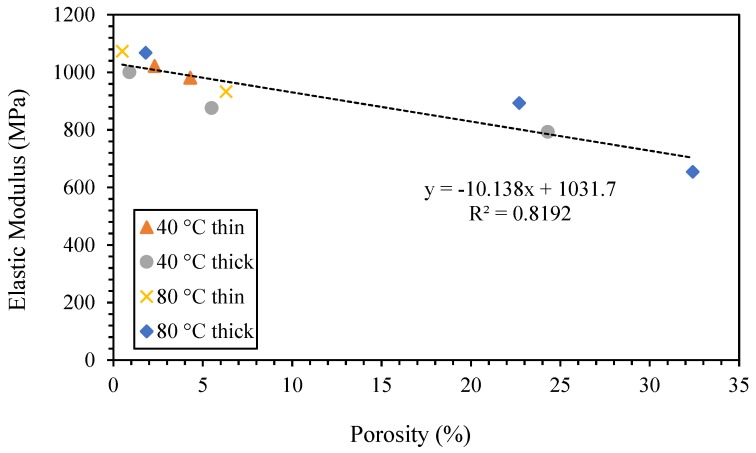
The correlation between the elastic modulus and porosity of the 3D printed samples.

The mechanical properties of the polished specimens (to separate the bottom, middle and top layers) were determined using a TMA in tensile mode. To better correlate the 3D printed samples’ morphology and mechanical properties, the solidity was calculated starting from the porosity (Equation (1)). Similar to the porosity, the solidity of almost all of the samples was higher than 90%, except for the middle and top layers of the thick samples. As expected, the porosity greatly influences the mechanical properties. Indeed, higher elastic moduli were measured for the bottom and top layers of the thin samples, while the lowest elastic modulus was that of the thick samples printed on the build-platform heated at 80 °C. However, in most of the cases, the elastic modulus was comparable to that of an injection-molded sample (Figure 4). Moreover, a solid elastic modulus can be defined, considering the ratio between the elastic modulus and solidity. For example, the middle layer of the thick sample printed on the build-platform heated at 40 °C had an elastic modulus of 792.7 MPa and a solidity of 75.7%. The solid elastic modulus of this sample is therefore 1047 MPa, which is not far from the elastic modulus of the injection-molded specimen (i.e., 1080 MPa). As a result, the average elastic modulus of the printed samples (1040 MPa) is about the same as that of the injection-molded sample. Finally, a linear correlation between the specimens’ elastic modulus and porosity was found (Figure 5).

**Figure 6:**
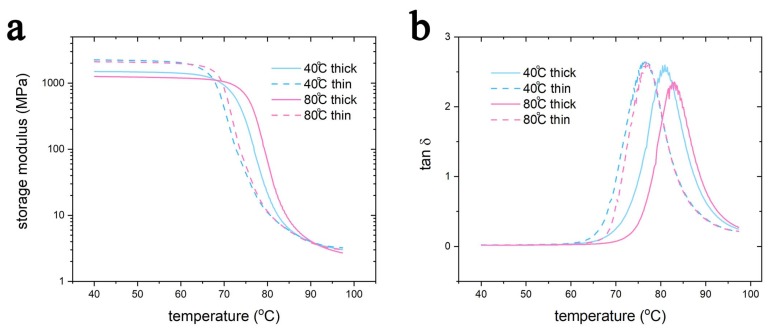
The DMA result of the samples. (**a**) storage modulus. (**b**) tan δ.

The thermo-mechanical properties were determined using a DMA, and the results are shown in Figure 6. Thinner samples have higher storage moduli (~2200 MPa) compared to thicker samples (~1350MPa) (Figure 6a) due to the influence of the porous morphology, as previously discussed. Moreover, the glass transition temperature (that can be considered to be the temperature corresponding to the tan δ peak) follows a similar trend, as is visible in Figure 6b. In particular, thinner samples have a slightly lower glass transition temperature (40 °C thin: 65 °C; 80 °C thin: 68 °C) compared to the thicker ones (40 °C thick: 68 °C; 80 °C thick: 72 °C). Finally, the samples printed on a build-platform heated at 40 °C have a lower glass transition temperature compared to the other set. However, the difference in the glass transition temperature was not so meaningful, and one of the goals of this study was to investigate if a heat treatment after 3D printing is a viable solution to eliminate this difference. 

### 3.3. XRD Results

**Figure 7:**
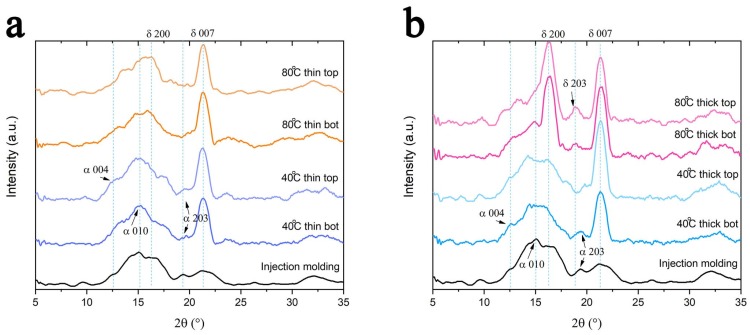
The XRD patterns of the (**a**) thin and (**b**) thick PLA samples.

Since PLA is a semi-crystalline polymer, it was important to monitor the change of crystallinity during a heat related processing method such as 3D printing. The different crystalline forms present in the printed object were studied using WAXD patterns (Figure 7). The results for thin and thick samples are reported in Figure 7a,b [28,30,48,49], respectively. For comparison, the result for the injection-molded sample was also presented. As evident, no significant differences can be recognized for the thin samples (Figure 7a). On the contrary, for the thick samples, an influence of the build-platform temperature was found (Figure 7b). In particular, the thick sample printed on a build-platform heated at 80 °C showed a very high δ (200) peak. As previously discussed, for PLA the α form is usually considered a stable form while the δ form is not [31]. This confirms why the thick samples printed on a build-platform heated at 80 °C have a lower elastic modulus than those printed on the build-platform heated at 40 °C (Figure 4).

**Figure 8:**
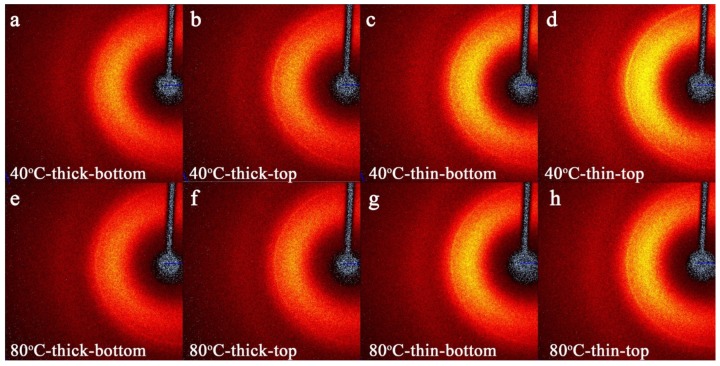
The 2D-XRD patterns for the (**a**) 40 °C-thick-bottom sample, (**b**) 40 °C-thick-top sample, (**c**) 40 °C-thin-bottom sample, (**d**) 40 °C-thin-top sample, (**e**) 80 °C-thick-bottom sample, (**f**) 80 °C-thick-top sample, (**g**) 80 °C-thin-bottom sample, and (**h**) 80 °C-thin-top sample.

**Figure 9:**
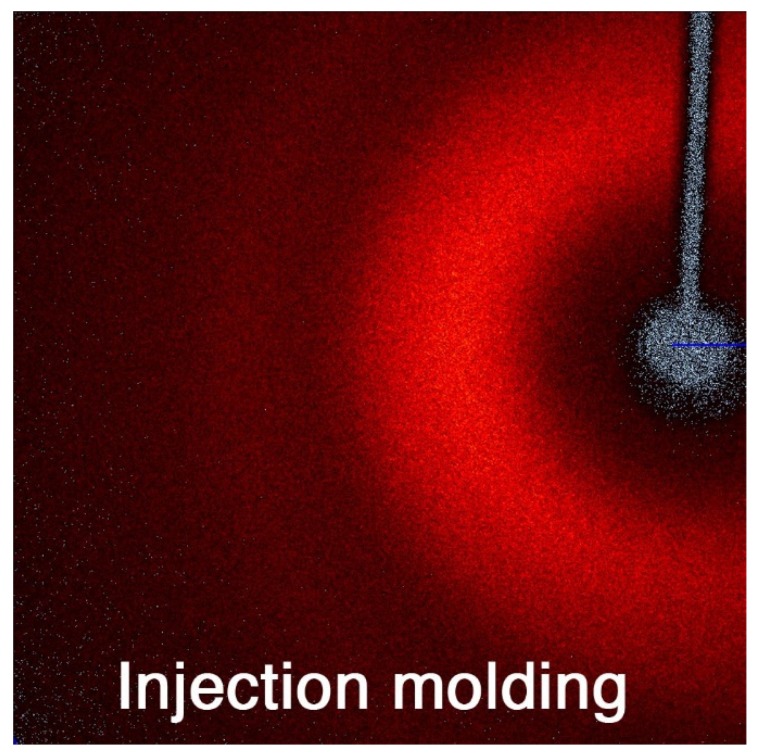
The 2D-XRD pattern for the injection molding sample.

In a typical 3D printing process, the PLA filament melts for several seconds in the extruder (i.e., the nozzle at a fixed temperature) before being extruded out. The extruding speed varies depending on the complexity of the printing path. The polymer chains are oriented during the extruding process inside the narrow nozzle due to the pressure drop [34]. As the crystallization speed of PLA is slower than the extruding process, it was important to control the build-platform temperature as well. In this case, we illustrated the orientation information in the printed PLA samples using the 2D-XRD method. As shown in Figure 8, all the samples show a certain degree of orientation (represented by the yellow arc and crest in the 2D-XRD patterns) influenced by the position of the layer (i.e., the distance from the build-platform) and the build-platform temperature. Theoretically, the degree of orientation can be calculated by measuring the arc span degree. However, due to the printed sample consisting of ±45° patterns, the calculated value could be highly inaccurate. In this case, only a qualitative analysis was carried out. For comparison, the 2D-XRD pattern of the injection-molded sample is shown in Figure 9. Compared to the isotropic sample (Figure 9), the sample printed at the low temperature build-platform (i.e., 40 °C) shows a higher degree of orientation (Figure 8a–d). A thin arc and thick crest shape pattern could be seen. The thin arc can be attributed to the δ (007) peak of Figure 7, while the crests are the peaks of α(004), α(010), δ(200) [28,30,48,49,50] and so on. A higher build-platform temperature results in less orientation than the other sample group because the polymer chains’ mobility is higher (being near T_g_), allowing for relaxation phenomena. Therefore, the orientation imposed during the filament deposition is partially lost. A thinner sample results in a much higher orientation degree because the sample preparation is faster than the other group and the resident time on the heated build-platform is lower. For this reason, the imposed orientation is not lost. The orientation degrees on both the top and bottom surfaces were similar. As a result, the printed semi-crystalline polymer could always be anisotropic due to orientation phenomena during the filament deposition, and this feature should be included when designing an object.

Comparing the XRD results with the mechanical properties, a correlation between the crystallinity and orientation of the printed part and mechanical properties, in both the static and dynamic tensile tests, could be found. A higher build-platform temperature reduces the orientation degree but improves the crystallization phenomena in thicker samples (Figure 7b) resulting in higher static moduli (Figure 4). On the contrary, a lower build-platform temperature preserves the orientation degree imposed during the filament deposition (Figure 8) giving higher storage moduli, especially for thin samples (Figure 6), i.e., dynamic mechanical properties.

### 3.4. Heat Treatment

**Figure 10:**
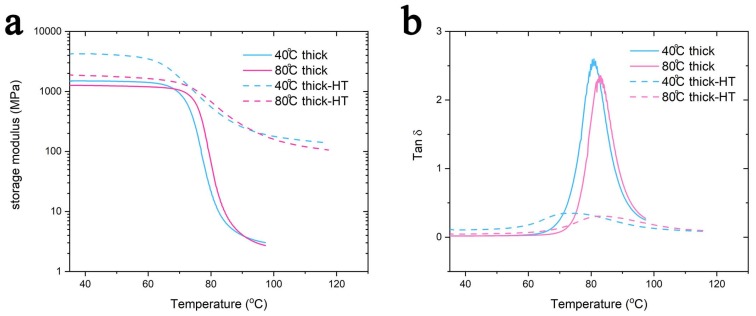
The DMA results of the thick samples before and after the heat treatment. HT is the abbreviation for heat treatment. (**a**) The storage modulus, and (**b**) tan δ.

**Figure 11:**
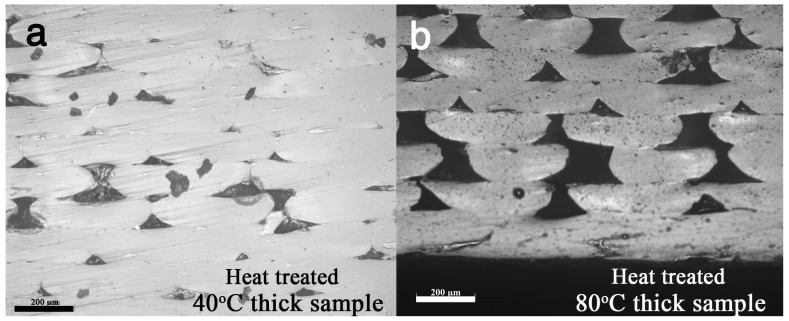
The cross section of the thick PLA samples printed on the build-platform heated at (**a**) 40 °C and (**b**) 80 °C (bar is 200 μm).

**Figure 12:**
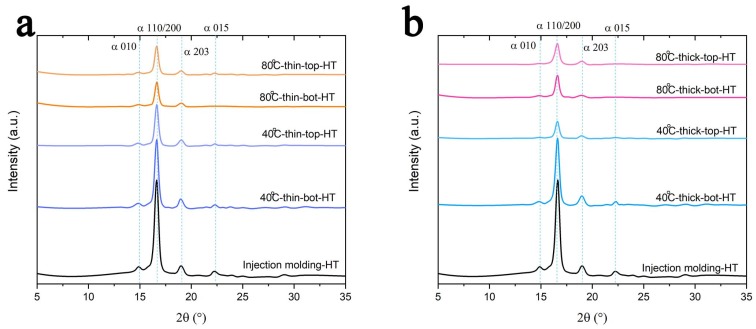
The XRD patterns of the heat treated (**a**) thin and (**b**) thick samples.

Heat treatment is a common method to reduce internal stress for newly built metallic and polymeric samples. As is well known in the additive manufacturing industry, internal stresses can accumulate during the printing process. Therefore, heat treatments can be considered as a routine procedure for printed samples, to reduce or remove internal stresses. However, the process should be well designed because an improper heat treatment could lead to severe deformations. In this study, printed samples were annealed at 120 °C for 10 min and then left to cool down at room temperature in air. However, this heat treatment leads to severe distortion for the thin samples because the heating and cooling procedures allow PLA to go through a recrystallization process without melting. Therefore, only thick samples were tested using DMA in a temperature ramping test, and the results are compared in Figure 10a. Heat treatment increased the samples’ modulus, especially at temperatures above 80 °C. The difference in modulus clearly shows that the heat treatment procedure changes the structure of the samples. The tan δ peak height and peak area (Figure 10b) of the two heat treated samples are lower than those of the original 3D printed samples because the elastic component increases more than the viscous component, indicating a decrease in the mobility of the polymer chains, attributed to an increase in the degree of crystallinity. As previously stated, the two factors may affect the mechanical property of the printed PLA: porosity and crystallinity. Figure 11 shows the cross section of the heat-treated parts, and obviously no porosity change occurred because annealing was done at 120 °C and the melting temperature of PLA is about 150 °C. This means that the modulus improvement derives from the other parameter, that is crystallinity. PLA has a cold crystallization temperature of approximatively 100 °C [51]. As we had a heat treatment at 120 °C, the cold crystallization of PLA was considered to be completed. In order to verify the change in crystallinity, XRD measurements for all the heat-treated samples were carried out (Figure 12). All the heat-treated samples have similar XRD patterns, and only α peaks can be identified [30,48,50]. This result not only confirms the crystallinity change in the heat-treated samples but also shows that all δ form crystallites change to α crystallites after the heat treatment. 

## 4. Conclusions

3D printing of semi-crystalline polymers is an emerging topic in the additive manufacturing industry thanks to the numerous offered advantages: low price, rapid tooling, and ease of use, among others. However, a trial and error approach is generally used to find the optimal printing parameters, and most of the attention is focused on the printing pattern and 3D printing object quality. Furthermore, one of the major concerns related to the 3D printing of polymers is the printed parts’ anisotropy. In this study, the influence of porosity and crystallinity on the final properties of 3D printing PLA specimens was investigated. Morphological investigations of specimens’ cross-sections revealed the formation of numerous pores in the specimens’ cross-sections, particularly in the middle and top layers. Moreover, thinner samples and lower build-platform temperatures led to higher mechanical properties thanks to a higher degree of orientation (as determined by 2D-XRD patterns). In addition, lower mechanical properties were found when the instable δ crystalline form was present. However, the elastic moduli of almost all of the samples are comparable to that of the injection-molded sample (i.e., the isotropic one). A thermal treatment (annealing at 120 °C for 10 min) was investigated in order to remove internal stresses and change the crystallinity of the printed samples. As measured with DMA, an improvement of the storage modulus was obtained after a thermal treatment for thick samples printed on a build-platform heated at 40 °C. Moreover, the thermal treatment was effective in removing the δ form, as was evident from XRD patterns following the heat treatment.
