# Erratum: Effect of Porosity and Crystallinity on 3D Printed PLA Properties. *Polymers* 2019, *11*, 1487

**DOI:** 10.3390/polym12010142

**Published:** 2020-01-06

**Authors:** Yuhan Liao, Chang Liu, Bartolomeo Coppola, Giuseppina Barra, Luciano Di Maio, Loredana Incarnato, Khalid Lafdi

**Affiliations:** 1 Department of Chemical and Materials Engineering, University of Dayton, 300 College Park Drive, Dayton, OH 45469, USA; liaoy1@udayton.edu; 2 Department of Industrial Engineering (DIIN), University of Salerno, via Giovanni Paolo II, 132, Edificio E 84084 Fisciano SA, Italy; bcoppola@unisa.it (B.C.); gbarra@unisa.it (G.B.); lincarnato@unisa.it (L.I.); 3 Department of Mechanical Engineering, Northumbria University, Newcastle upon Tyne NE1 8ST, UK

The authors wish to make a change to the published paper [1]. In the original manuscript, there are mistakes on the scale bar of Figures 2 and 3. The unit of the scale bar should be “μm”, not “nm”. The corrected Figures 2 and 3 are presented below.

**Figure 2:**
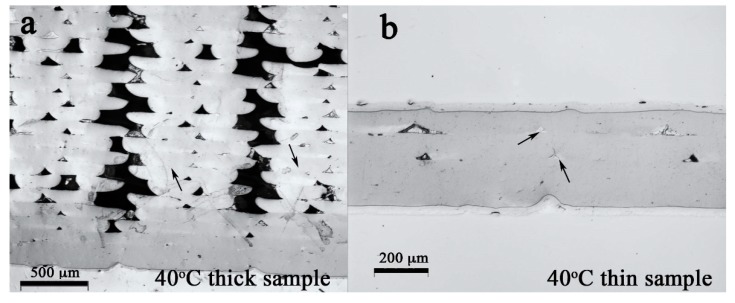
The cross section of the (**a**) thick and (**b**) thin PLA samples printed on the build-platform heated at 40 °C. The bar in (**a**) is 500 μm. The bar in (**b**) is 200 μm.

**Figure 3:**
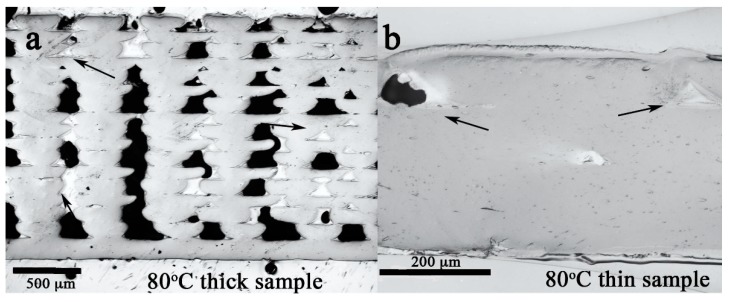
The cross section of the (**a**) thick and (**b**) thin PLA samples printed on the build-platform heated at 80 °C. The bar in (**a**) is 500 μm. The bar in (**b**) is 200 μm.

The authors apologize for any inconvenience caused and the change does not affect the scientific results. The manuscript will be updated, and the original will remain online on the article webpage at https://www.mdpi.com/2073-4360/11/9/1487.
